# Synthetic Mono-Rhamnolipids Display Direct Antifungal Effects and Trigger an Innate Immune Response in Tomato against *Botrytis Cinerea*

**DOI:** 10.3390/molecules25143108

**Published:** 2020-07-08

**Authors:** Mathilde Robineau, Sarah Le Guenic, Lisa Sanchez, Ludovic Chaveriat, Vincent Lequart, Nicolas Joly, Maryline Calonne, Cédric Jacquard, Stéphane Declerck, Patrick Martin, Stephan Dorey, Essaid Ait Barka

**Affiliations:** 1 RIBP-EA 4707, SFR Condorcet FR CNRS 3417, University of Reims Champagne-Ardenne, 51100 Reims, France; mathilde.robineau@univ-reims.fr (M.R.); lisa.sanchez@univ-reims.fr (L.S.); cedric.jacquard@univ-reims.fr (C.J.); 2 UnilaSalle, Unité Transformations & Agroressources, Université d’Artois, ULR7519, F-62408 Béthune, France; sarah.leguenic@gmail.com (S.L.G.); ludovic.chaveriat@univ-artois.fr (L.C.); vincent.lequart@univ-artois.fr (V.L.); nicolas.joly@univ-artois.fr (N.J.); patrick.martin@univ-artois.fr (P.M.); 3 Earth and Life Institute, Applied Microbiology, Mycology, Université catholique de Louvain, Croix du Sud, 2 box L7.05.06, 1348 Louvain-la-Neuve, Belgium; maryline.calonne@uclouvain.be (M.C.); stephan.declerck@uclouvain.be (S.D.)

**Keywords:** innate immunity, tomato, elicitors, *Botrytis*, induced resistance

## Abstract

Natural rhamnolipids are potential biocontrol agents for plant protection against bacterial and fungal diseases. In this work, we synthetized new synthetic mono-rhamnolipids (smRLs) consisting in a rhamnose connected to a simple acyl chain and differing by the nature of the link and the length of the lipid tail. We then investigated the effects of these ether, ester, carbamate or succinate smRL derivatives on *Botrytis cinerea* development, symptoms spreading on tomato leaves and immune responses in tomato plants. Our results demonstrate that synthetic smRLs are able to trigger early and late immunity-related plant defense responses in tomato and increase plant resistance against *B. cinerea* in controlled conditions. Structure-function analysis showed that chain length of the lipidic part and type of acyl chain were critical to smRLs immune activity and to the extent of symptoms caused by the fungus on tomato leaves.

## 1. Introduction

The surveillance system of plant immunity relies on the perception by plant IP receptors (IPRs) of invasion patterns (IPs), also known as elicitors, to trigger plant defense responses [1,2,3,4]. Signaling responses of IP-triggered immunity include the production of reactive oxygen species (ROS), ion fluxes, and activation of cytoplasmic protein kinases including mitogen-activated protein kinases (MAPKs) and Ca^2+^-dependent protein kinases (CDPKs) [5,6,7]. Plant signaling hormones including among others, salicylic acid (SA), jasmonic acid (JA) and ethylene (ET) regulate transcriptional reprogramming, leading to plant defense gene activation [8]. The plant immune response results in the strengthening of cell walls and production of antimicrobial compounds [9].

Invasion patterns from microbial origin are represented by structurally distinct molecules, including well known components such as flagellin peptides, medium-chain 3-hydroxy fatty acids, chitin, β-glucans and amphiphilic compounds such as rhamnolipids and lipopeptides [3,10]. Synthetic elicitors are small compounds, structurally distinct from natural IPs or chemically inspired by IPs [3,11]. They can trigger the plant immune response by mimicking IP perception or IP-triggered plant signaling. Most of these molecules can induce plant resistance against pathogens [11]. To date, several synthetic elicitors have been characterized, including low-molecular-weight polyacrylic acid derivatives, imprimatins, sulfonamides, adipic acid derivatives or SA and JA analogs [11]. 2,6-dichloro-isonicotinic acid (INA) and benzo(1,2,3)thiadiazole-7-carbothioic acid S-methyl ester (BTH) (also known as Bion^®^) have been reported to mimic SA-triggered immune responses [12,13,14,15]. 2-(5-bromo-2-hydroxy-phenyl)-thiazolidine-4-carboxylic acid (BHTC) have also been shown to induce plant disease resistance against bacteria, oomycetes, and fungi [16]. 3,5-dichloroanthranilic acid (DCA) induces NPR (non expressor of pathogenesis-related genes) 1-dependent and NPR1-independent mechanisms of disease resistance against the pathogenic oomycete *Hyaloperonospora arabidopsidis* and the bacterial pathogen *Pseudomonas syringae* pv. *tomato* DC3000 (Pst) in *Arabidopsis thaliana* [17]. DPMP (2,4-dichloro-{(E)-[(3-methoxyphenyl)imino]methyl} phenol) has also been reported to trigger a robust immune response in Arabidopsis and tomato [18]. Synthetic amphiphilic molecules including short cationic lipopeptides [19], lipid 3-tetradecylamino-tert-butyl-*N*-tetradecylpropionamidine (diC14) [20] and more recently, bolaform rhamnolipids [21] can also stimulate the plant immune system.

In the last few years, attention to amphiphilic molecules increased because of their multiple applications in different areas including bioremediation, pharmacology or agriculture [21]. Rhamnolipid surfactants are well known for their biocompatibility, biodegradability or low toxicity to non-target organisms [22,23,24]. These molecules have direct antimicrobial properties against various fungal species in vitro and in vivo [25,26,27,28,29,30]. A number of studies have also reported that natural rhamnolipids are able to stimulate plant immunity and trigger induced resistance against various phytopathogens [31,32,33,34].

In the present work, the objective was to investigate the potential of bio-inspired synthetic mono-rhamnolipids (smRLs) on inhibiting the growth of *Botrytis cinerea* and/or inducing tomato immune responses. The list of smRLs used in this study were listed as Supplementary Materials (Table S1). Taken as a whole, the structure/function analysis revealed that the nature of the acyl chain is determinant in the activation of the immune response in tomato. Our results suggest that smRLs with acyl chain length ranging from 10 to 12 carbons are perceived by tomato, inducing the plant defence mechanisms. smRLs also have a direct antifungal effect by both altering *Botrytis* conidial spore germination and mycelial development.

## 2. Results

### 2.1. Synthesis of smRLs

Naturals Rhamnolipids (RLs) are amphiphilic molecules typically composed of a hydrophobic alkyl chain linked through a glycosidic bond to mono- or di-rhamnoses. To obtain a first series of bio-inspired smRLs, we have synthesized easily accessible derivatives: RLs ethers analogues. Alkylated carbohydrates on anomeric position have been widely described in the literature [35] but rarely those synthesized from rhamnose [36,37].

**Scheme 1:**
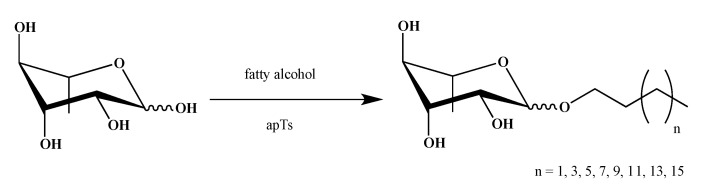
Synthesis of rhamnose ethers (Eth-C4 to Eth-C16).

Rhamnose ether derivatives were obtained from the reaction between l-rhamnose and a fatty alcohol with various chain lengths (4 to 18 carbon atoms), without solvent and in the presence of *p*-toluenesulfonic acid as catalyst [38] with a yield of 42–64% (Scheme 1).

**Scheme 2:**
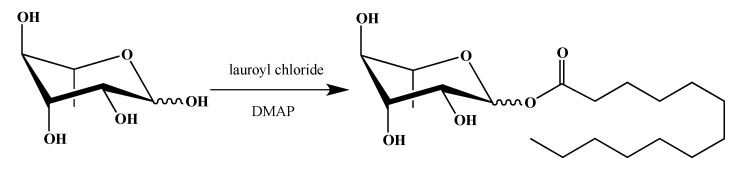
Synthesis of rhamnose ester (Est-C12).

C12-derived molecules with variable glycosidic bond were then synthesized to conduct the smRLs structure-function study. To stay close to the structure of the natural RLs, esterification on the anomeric position of l-rhamnose has been carried out (Scheme 2). Acyl derivatives were obtained by esterification of l-rhamnose with dodecanoyl chloride in *N*,*N*-Dimethylformamide (DMF) in the presence of *N*,*N*-Dimethylpyridin-4-amine (DMAP) [39] with a 54% yield.

**Scheme 3:**
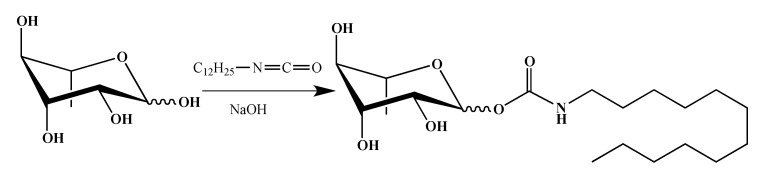
Synthesis of rhamnose carbamate (Car-C12).

In the literature, glycoside derivatives with a carbamate linker were described as potential pesticide or fungicide compounds [40]. Thus, to add these potential properties to our RLs derivative, the synthesis of a rhamnose carbamate was developed (Scheme 3). Carbamate derivative was obtained by reaction between dodecyl isocyanate and l-rhamnose in basic medium [41] with a 48% yield.

**Scheme 4:**
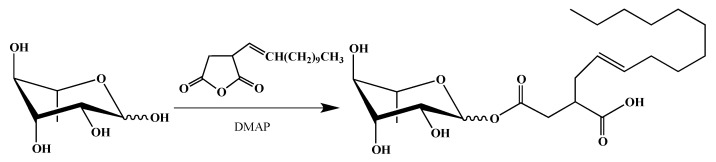
Synthesis of rhamnose succinate (Suc-C12).

Finally, we wanted to mimic as much as possible natural RLs. Most of them are described as compounds with a hydroxyfatty acid branched alkyl chain linked to the carbohydrate moiety by an ester group [42,43]. Therefore, we decided to use a commercially available (dodecenyl)succinic anhydride, already used to modify surfactant properties of carbohydrate polymers [44,45] but not those of rhamnose. Using similar reaction conditions to obtain an ester derivative, mono-rhamnosyl (dodecenyl) succinate was isolated, as described in the Scheme 4 with a 54% yield:

### 2.2. Ester, Ether, Succinate and Carbamate-Derived smRLs Differentially Activate Plant Immune Signalling in Tomato Leaves

**Figure 1:**
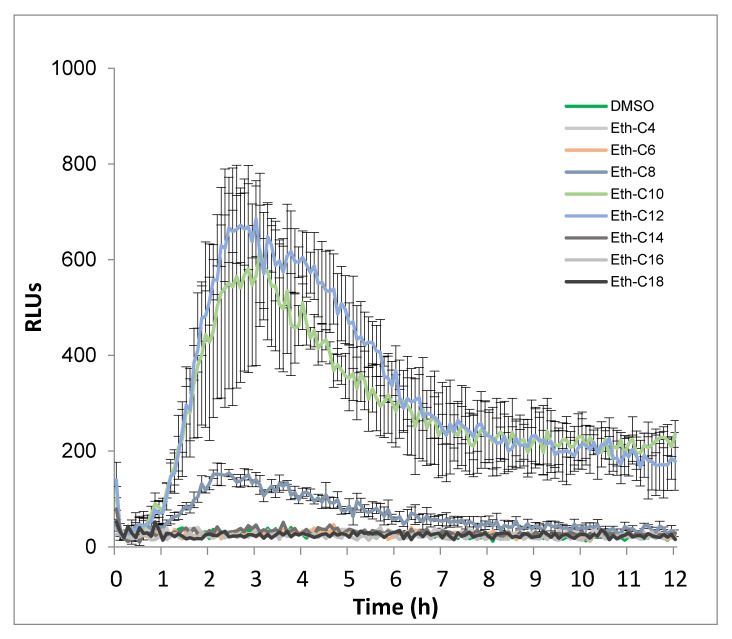
Production of reactive oxygen species (ROS) in tomato leaf disks after treatment with ether-synthetic mono-rhamnolipids (Eth-smRLs) exhibiting different carbon chain lengths at 100 µM. Dimethyl sulfoxide (DMSO; 0.1%) was used as control. ROS production was measured using the chemiluminescence of luminol and photon counts were expressed as relative luminescence units (RLUs).

We firstly investigated whether the simplest smRLs, with an acyl chain coupled to rhamnose by an ether link (Eth-smRLs) could activate an immune response in tomato. Tomato leaves were challenged with Eth-smRLs with carbon chain lengths varying from 4 to 18 carbons, at 100 µM (a concentration already used for synthetic rhamnolipid bolaformes by Luzuriaga-Loaiza et 2018 [21]. ROS production, a classical marker of the plant immune response, was monitored over a 12 h time course. Our results indicated that Eth-smRL-triggered ROS production is dependent on the length of the carbon chain (Figure 1 and Figure S1). Indeed, ROS production was not affected by C4-, C6-, C14-, C16- and C18-derived molecules, and was slightly induced by C8. By contrast, Eth-C10 and Eth-C12 triggered a strong and long-lasting ROS production in tomato leaves, with a maximum of production 3 h after treatment. However, Eth-C12 was the most active molecule. Eth-smRL-triggered ROS production is also dose-dependent. Indeed, dose–response experiments confirmed that although ROS production could be observed at 50 µM, the optimal concentration inducing strong and consistent ROS responses in tomato was 100 µM (Figure S2). This concentration was, therefore, used for the next experiments in planta. 

**Figure 2:**
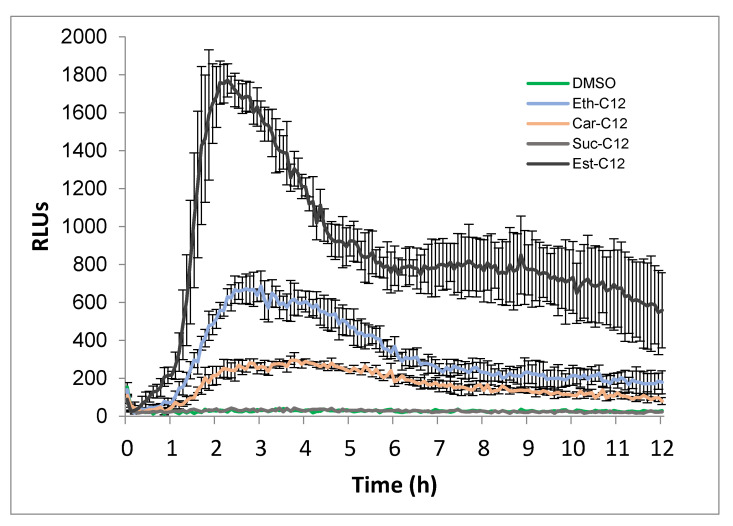
ROS production after smRLs treatment of tomato leaf disks. smRLs with carbamate (Car), succinate (Suc), ester (Est) or ether (Eth) links between the rhamnose and the acyl chain or DMSO (0.1%) control were used in these experiments. Reactive oxygen species (ROS) production was measured using the chemiluminescence of luminol and photon counts were expressed as relative luminescence units (RLUs).

Next, we investigated the influence of the link between the rhamnose and the acyl chain in the eliciting activity. smRLs-derived molecules including carbamate (Car), succinate (Suc), ester (Est) were monitored for ROS responses. Because a 12-carbon-length tail was found to be the most active conformation (Figure 1), the following experiments were achieved using C12-derived molecules. Est-C12 was the most active compound to induce ROS production in tomato leaves with a stronger burst of ROS than Ether-C12 (Figure 2). On the other hand, a small but significant increase in ROS production was also triggered after Car-C12 application, while no response was observed with Suc-C12 (Figure 2). 

### 2.3. smRLs Inhibit Conidia Germination and Mycelium Growth of Botrytis Cinerea

#### 2.3.1. Conidial Germination

**Figure 3:**
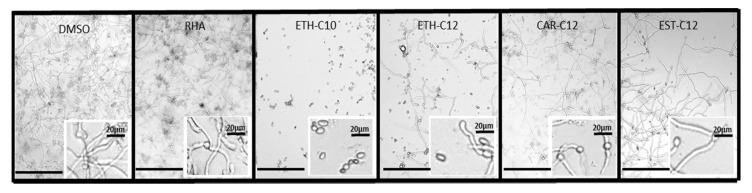
Effect of smRLs at 100 µM on *Botrytis cinerea* conidial germination 24 hai observed by inverted microscopy. Treatment with DMSO (0.1%) and natural rhamnolipids mix (RHA) served as negative and positive control, respectively. All (sm)RLs were tested at 300 µM. Scale bars correspond to 275 µm.

Natural RLs are known to have direct antimicrobial activities against fungi and zoosporic plant pathogens [43]. In addition, these RLs are able to affect conidial germination and mycelial growth of the necrotrophic fungus *B. cinerea* [32,33,34]. Based on their eliciting activities, four smRLs including Eth-C10, Eth-C12, Car-C12, and Est-C12 were selected for the following experiments. The formation and development of the *B. cinerea* germ tube were monitored 24 h after incubation of conidia with the four smRLs at 300 µM (Figure 3).

Most conidia germinated and hyphae were fully developed in the control (DMSO). In presence of Eth-C10, conidial germination was totally inhibited. Interestingly, with Eth-C12, conidial germination was highly prevented, but the effect was less pronounced compared to Eth-C10. In the presence of Car-C12 and Est-C12, the few germinated conidia presented fully developed hyphae. All the molecules were more effective than the natural rhamnolipid mix on this specific strain of *B. cinerea*. These results demonstrate the direct antifungal activity of all smRLs on *B. cinerea* but with differential efficiencies.

#### 2.3.2. Mycelium Growth

**Figure 4:**
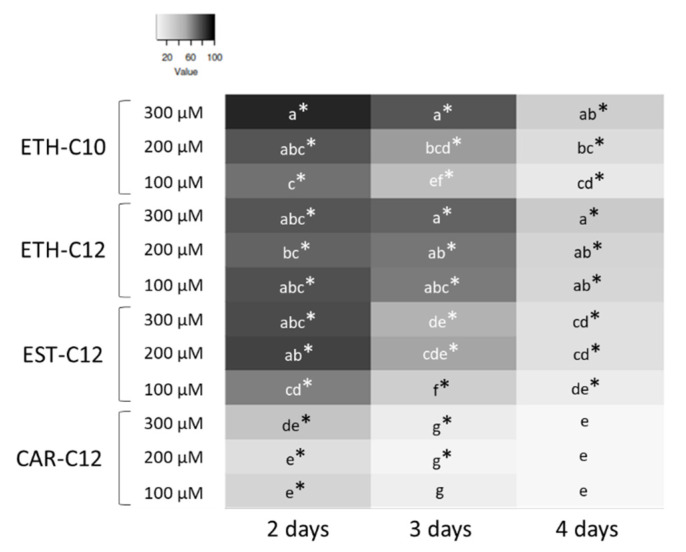
Heat map of *B. cinerea* growth inhibition (%—in comparison with the control DMSO 0.1%) after 2, 3 and 4 days of growth in presence of smRLs (Eth-C10, Eth-C12, Est-C12 and Car-C12) added in wells of microplates before conidia inoculation. In each column, * indicates significant growth inhibition in comparison to the DMSO 0.1% control, and lower-case letters indicate significant difference among the presented means, according to an ANOVA followed by a Tukey-HSD post-hoc test (*p* ≤ 0.05).

The formation and subsequent development of the mycelium from germinated conidia of *B. cinerea* were monitored over 3 days after conidia incubation with the four smRLs at 100, 200 and 300 µM (Figure 4).

**Figure 5:**
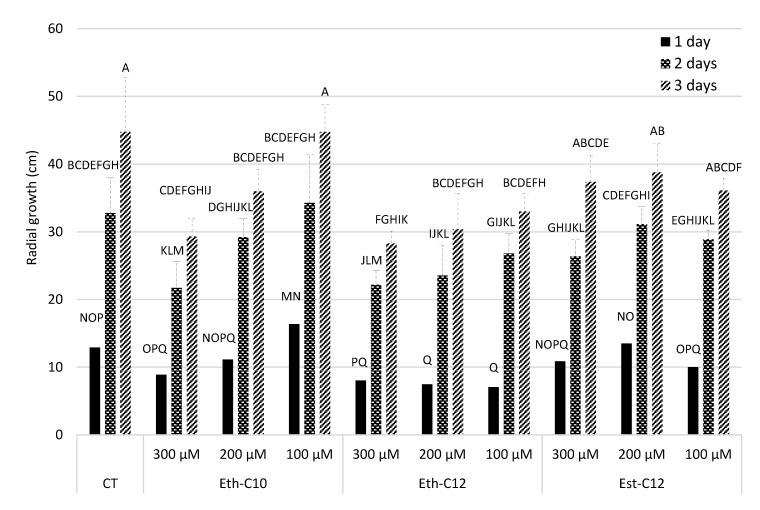
Radial growth surface (cm²) of a mycelium of *B. cinerea* growing for 1 day evaluated after 1, 2 and 3 days of growth in absence (CT-DMSO 0.1%) or in presence of smRLs (Eth-C10, Eth-C12 and Est-C12). Different letters indicate significant difference among all the means, according to a repeated-measures ANOVA followed by a Tukey-HSD post-hoc test (*p* ≤ 0.05).

When the solid medium was supplemented with smRLs before inoculation with conidia of *B. cinerea*, all treatments significantly reduced in vitro growth of the pathogen after 2 days (Figure 4). After 3 and 4 days, the percentage of growth inhibition decreased for all the treatments, irrespective of the applied concentration. The greatest growth reduction was obtained with Eth-C10 and Eth-C12 at 300 µM. On the contrary, no or little reduction in growth was observed at 3 and 4 days with Car-C12. As Car-C12 did not show a strong effect on the conidial germination and subsequent mycelial growth, it was not considered for the dual-test on mycelium that had been actively growing for 1 day. The effects of Eth-C10, Eth-C12, and Est-C12 monitored on the growth of a mycelium that had been actively growing for 1 day are presented in Figure 5.

Our data showed a strong interaction between the treatments and the time of measurements (*p* = 0.0115), suggesting that growth of *B. cinerea* responded differently to the treatments according to the date of observation (after 1, 2 or 3 days of growth). In, general, Est-C12 did not significantly inhibit *B. cinerea* radial growth in comparison with the DMSO control, irrespective of the date of measurement or the concentration (Figure 5). The same observation could be made with 100 and 200 µM of Eth-C10, except after 3 days of growth in the presence of 200 µM, for which a slight reduction in radial growth was measured. Conversely, a strong radial growth reduction was obtained with 300 µM of Eth-C10 and with Eth-C12, especially after 2 and 3 days of growth. Altogether, our in vitro experiments showed that Eth-derived smRLs are the most active compounds in terms of antifungal effects. 

### 2.4. smRLs Differentially Stimulate Tomato Immunity against Botrytis Cinerea

**Figure 6:**
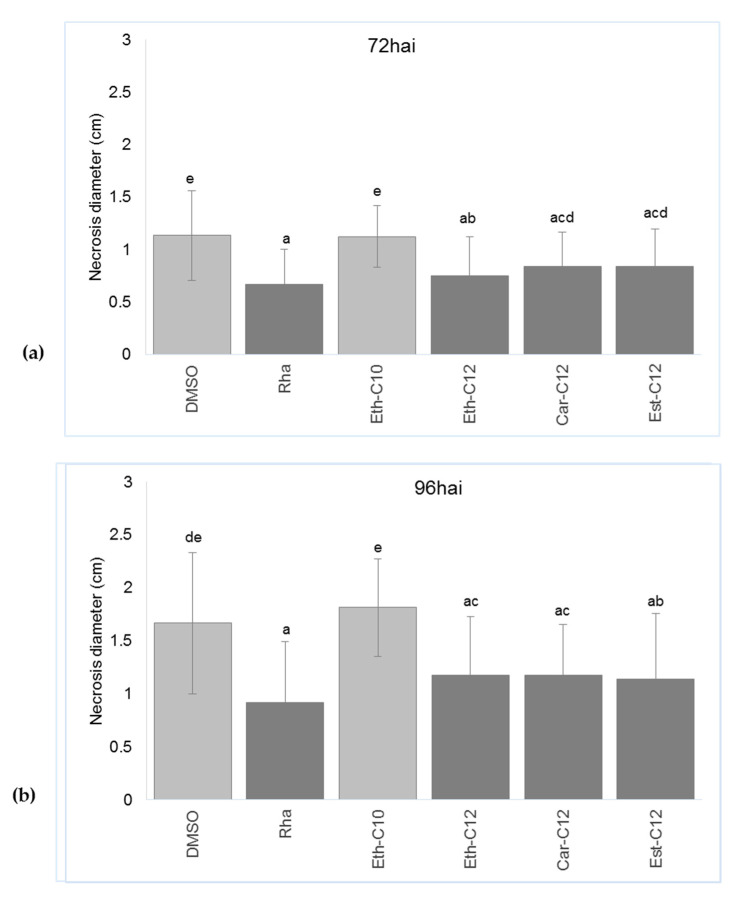
Impacts of smRLs on necrosis symptoms caused by *B. cinerea* on tomato leaves. Plants were sprayed with 300 µM of smRLs, natural rhamnolipid mix (Rha) or DMSO 0.1% and 3 days later, detached leaves were inoculated with the fungus. Necrosis diameter measures (**a**) 72 hai; (**b**) 96 hai. Values shown are means +/− SD of three independent repetitions (each repetition was realized in triplicate and each replicate consisted of four leaves). Different lower-case letters indicate significant differences between the treatments, as determined by an ANOVA followed by the Tukey-HSD post-hoc test (*p* ≤ 0.05).

**Figure 7:**
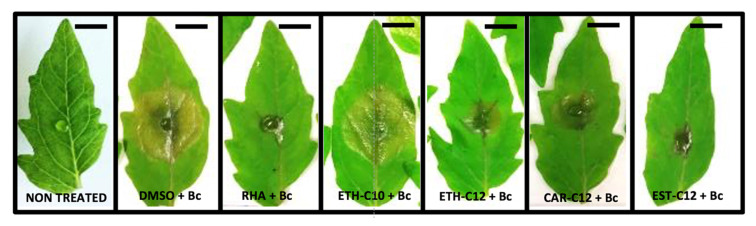
Pictures of symptoms development of *B. cinerea* on detached leaves treated with different smRLs. Plants were sprayed with 300 µM of smRLs, natural rhamnolipid mix (Rha) or DMSO 0.1% and 3 days later, detached leaves were inoculated with the fungus (one droplet of 10 µL in the center of the leave). Non-treated leaves with one droplet of PDB were used as control. Scale bars correspond to 1 cm.

Three days before *B. cinerea* infection, tomato plantlets were sprayed with Eth-C10, Eth-C12, Car-C12 or Est-C12 smRLs at 300 µM and infection by *B. cinerea* was monitored by measuring necrosis diameter 72 and 96 h after inoculation (hai) (Figure 6 and Figure 7).

Tomato leaves treated with 0.1% DMSO presented a high level of infection 72 and 96 hai with 1.1 cm and 1.6 cm necrosis diameters, respectively (Figure 6 and Figure 7). No reduction of symptoms was observed with Eth-C10, while a significant reduction of symptoms was observed with Eth-C12, Car-C12 and Est-C12 as compared to the control 72 and 96 hai (Figure 6 and Figure 7).

### 2.5. smRLs that Decrease Symptoms Development by B. cinerea on Detached Tomato Leaves Potentiate Their Defence Responses

**Figure 8:**
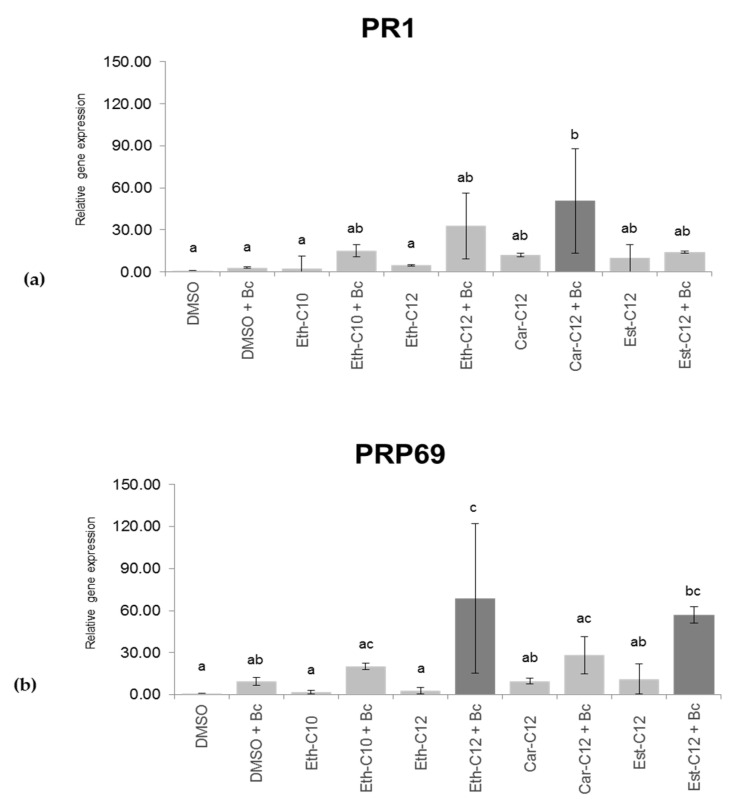
Relative defense gene expression in detached tomato leaves treated with only smRLs (300 µM) or smRLs (300 µM) and *B. cinerea* (Bc) 3 days post-elicitation, 48 h after infection with the pathogen. (**a**) Expression level of *PR1*; (**b**) Expression level of *PRP69*. Different lower-case letters indicate significant differences among the means as determined by an ANOVA followed by the Tukey-HSD post-hoc test (*p* ≤ 0.05). Fold expression values are normalized against tomato *EF1α* and *Actin* genes as controls and DMSO 0.1% as a reference sample.

To investigate whether smRLs activate the defense gene and/or a priming response, the expression of two well-known defense markers in tomato, PR1 and PRP69 (Supplementary Materials, Table S1) [46,47] was evaluated on leaves 120 h after smRLs spaying and 48 h after *B. cinerea* challenge. In the absence of *B. cinerea*, no statistically significant induction of gene expression was revealed after treatment with Eth-C10, Eth-C12, Car-C12 or Est-C12 (Figure 8). Under priming conditions, Car-C12 significantly stimulated PR1 expression but did not affect PRP69 activation. Eth-C12 and Est-C12 significantly primed PRP69 gene expression after *B. cinerea* challenge (Figure 8b). These results suggest that smRLs could differentially modulate the plant priming responses after pathogen challenge, as exemplified by PR1 and PRP69 gene expression.

## 3. Discussion

In this study, we demonstrated that ester, ether, succinate and carbamate-derived smRLs are differentially perceived by tomato cells and induce an immune response characterized by a late signaling ROS production, and for some of them, a potent priming response. Several synthetic compounds produced by combinatorial and organic chemistry have recently been identified as efficient elicitors [11]. All these compounds induce disease resistance to pathogens, at least in controlled conditions. However, their mode of action can strongly differ depending on the structures of the molecules. For instance, DCA induced fast, strong, and transient disease resistance against the pathogenic oomycete *Hyaloperonospora arabidopsidis* and the bacterial pathogen Pst [17]. Nevertheless, the DCA-induced resistance to *H. arabidopsidis* was only partially dependent on the NPR1 pathway [17]. Also, a synthetic cationic lipid diC14 with amphiphilic properties has been reported to protect plants against Pst, but not against *B. cinerea,* and the induced resistance to Pst was SA-dependent [20]. Recently, rhamnolipid bolaforms, particularly with a 14-carbon chain length, have been shown to trigger a SA-dependent immune response in Arabidopsis against Pst [21].

Interestingly, synthetic elicitors characterized to date are efficient at relatively high concentrations (generally from 100 µM to 1 mM) compared to canonical IPs such as flagellin, active at micromolar or even nanomolar concentrations [48]. Similarly, smRLs were reported to induce defense reactions such as ROS production at 100 µM but not at 25 µM. Interestingly, the same concentrations were effective for rhamnolipid bolaforms (SRBs). In particular, several hundred µM of the molecules were needed to activate the plant local induced resistance [21]. These elevated concentrations might be explained by a hormone-like mode of action or a different perception mechanism in which classical plant receptors are not involved. In addition, because these compounds are sprayed on the leaf surface, it cannot be excluded that only a fraction of the molecules reached leaf mesophyll and the plant cell membrane.

To our knowledge, little data exist on signaling events involved in synthetic elicitor-triggered immunity. Here, we demonstrated that smRLs induced a strong ROS production as observed with SRBs [21]. smRL-induced atypical ROS response with a long-lasting peak, could suggest that these compounds interact with the lipids from the plant plasma membrane as it has been suggested for other synthetic rhamnolipids [21,49]. Regarding the cell perception of smRLs, we have demonstrated that the chemical structure of smRLs, in particular the length and nature of the acyl chain, is important for plant perception and induction of defense signals. The most active smRLs were characterized by a C12 acyl chain. Acyl chains of 10 and 12 carbons induced an effective elicitation in contrast to C14, C16 or C18, which were inactive or showed a very low eliciting activity. At the opposite, synthetic ultrashort cationic lipopeptides with chains containing 16 carbons have been reported to be very active [19]. In addition, diC14 was more efficient in inducing plant innate immunity than diC16 [20]. Lipopeptides such as surfactins have been reported to display an immune response depending on the concentration and/or the length of the fatty acid chain [50,51]. Recently, some data showed that mono-alkyl lipophilic cations (MALCs) with amphipathic properties could activate an immune response and effectively protects cereals against *Septoria tritici* blotch and rice blast disease [52]. Interestingly, only C18-SMe_2_^+^ with an 18-carbon chain length, and not C12-G^+^ nor C18-NMe_3_^+^ could trigger a strong plant ROS production, suggesting that chain length and the polar head could influence the response. Accordingly, we found that both the length of the fatty acid tail but also the nature of the bonds in the molecules influenced both the ROS response and increased resistance in plant in controlled conditions.

RLs were previously described for their direct antifungal effect [25,30,32,53,54,55]. In the present study, a similar effect of smRLs was demonstrated against *B. cinerea* through an inhibition of conidial germination as well as an alteration of mycelial growth. Moreover, we also found a curative effect of Eth-C12. Both preventive and curative effects have also been reported for natural RLs [31], exemplifying a dual role of rhamnose-based glycolipids as antifungal agents. Interestingly, MALCs also exhibited antifungal properties, mainly through inhibition of fungus oxidative phosphorylation and fungus apoptosis [52]. To date, we do not know how smRLs act to alter fungal germination and growth but given their amphiphilic properties it can be assumed that they interfere with the fungus plasma membrane as has been postulated for natural RLs [29].

We can hypothesize that smRLs such as Eth-C12, Car-C12 or Est-C12 might increase resistance of tomato against *B. cinerea* by combining a direct antimicrobial effect and induction of plant defense mechanisms as has previously been shown for natural RLs [33] and recently suggested for MALCs [52]. This new type of elicitor, with a dual mode of action combining antifungal properties and stimulation of plant innate immunity, offers promising avenues that could participate in broadening the variety of crop protection products available in the agriculture market. Moreover, when compared to the complicated and high cost of microbial elicitors’ purification, chemical synthesis is easy to scale-up at industrial level with affordable costs. Further, it provides the additional advantage of making a subsequent structural modification possible, in order to adapt the final product to new uses and to increase their activities in plant protection.

## 4. Materials and Methods 

### 4.1. smRLs Synthesis 

The conversions and purities of the products were determined by NMR spectroscopy. NMR spectra were recorded on a Bruker DRX300 spectrometer (Bruker Biospin, France) operating at 300 MHz for ^1^H nuclei and 75 MHz for ^13^C nuclei. CDCl_3_ (99.50% isotopic purity) were purchased from Euriso-Top (Saarbrücken, Germany). The progress of the reactions was checked by thin-layer chromatography (TLC) on Merck silica gel 60 glass plates. Detection was carried out by spraying the chromatograms with 10% sulfuric acid in ethanol and heating them to 100 °C. Flash column chromatography was performed with silica gel (40–100 lm, Merck, Molsheim, France): all chemicals were of reagent grade and used without further purification.

#### 4.1.1. Rhamnose Ethers

l-rhamnose (3.0 g, 18.3 mmol, 1 eq.), fatty alcohol (C4–C18, 36.6 mmol, 2 eq.), and para-toluene sulfonic acid (0.35 g, 1.8 mmol, 0.1 eq.) were stirred at 70 °C. After 24 h, the reaction was cooled, and neutralized with solid NaHCO_3_. The organic layer was extracted with chloroform, dried with MgSO_4_ and concentrated under reduced pressure. The rhamnose ether was isolated after purification by column chromatography (EtOAc/Petroleum spirit 7:3) with 30–69% yields.

##### Butyl α/β-l-Rhamnopyranoside

Colorless oil (β/α ratio 3:7, 69%); Rf 0.56 (AcOEt/MeOH 9/1); ^1^H NMR (CDCl_3_): 4.72 (m, 2H), 3.89 m, 2H), 3.75–3.71 (m, 2H), 3.67–3.58 (m, 4H), 3.44–3.34 (m, 4H), 1.58–1.53 (m, 4H), 1.40–1.24 (m, 10H), 0.86 (m, 6H, CH_3_) ^13^C NMR (CDCl_3_): δ 100.1, 97.9, 73.4, 72.6, 71.5, 71.2, 71.1, 69.9, 69.7, 68.5, 31.4–22.0 (CH_2_ alkyl chain), 18.0, 14.1, 14.0.

##### Hexyl α/β-l-Rhamnopyranoside

Colorless oil (β/α ratio 2:8, 52%); Rf 0.54 (AcOEt/MeOH 9/1); ^1^H NMR (CDCl_3_): 4.72 (m, 2H), 3.89 (m, 2H), 3.76–3.72 (m, 2H), 3.66–3.59 (m, 4H), 3.47–3.34 (m, 4H), 1.59–1.50 (m, 4H), 1.29 (m, 18H), 0.87 (m, 6H, CH_3_) ^13^C NMR (CDCl_3_): δ 100.4, 98.2, 73.5, 72.6, 71.4, 71.1, 70.8, 69.9, 69.7, 68.3, 31.2–21.9 (CH_2_ alkyl chain), 17.5, 14.2.

##### Octyl α/β-l-rhamnopyranoside

Yellow oil (β/α ratio 2:8, 38%); Rf 0.52 (AcOEt/MeOH 9/1); ^1^H NMR (CDCl_3_): 4.67 (m, 2H), 3.84 (m, 2H), 3.70-3.66 (m, 2H), 3.58–3.52 (m, 4H), 3.3–3.30 (m, 4H), 1.46–1.51 (m, 4H), 1.24–1.21 (m, 26H), 0.82 (m, 6H, CH_3_) ^13^C NMR (CDCl_3_): δ 100.2, 98.7, 73.4, 72.4, 71.4, 71.1, 70.8, 69.9, 69.7, 68.0, 32.0–22.8 (CH_2_ alkyl chain), 17.7, 14.3.

##### Decyl α/β-l-rhamnopyranoside 

Light yellow solid (β/α ratio 3:7, 35%); Rf 0.53 (AcOEt/MeOH 9/1); ^1^H NMR (CDCl_3_): 4.72 (m, 2H), 3.89 (m, 2H), 3.76–3.73 (m, 2H), 3.65–3.58 (m, 4H), 3.45–3.33 (m, 4H), 1.57–1.52 (m, 4H), 1.30–1.25 (m, 34H), 0.87 (m, 6H, CH_3_) ^13^C NMR (CDCl_3_): δ 100.1, 98.8, 73.5, 72.4, 72.0, 71.2, 70.7, 69.8, 68.0, 32.0–22.8 (CH_2_ alkyl chain), 17.9, 17.7, 14.3.

##### Dodecyl α/β-l-Rhamnopyranoside 

White solid (β/α ratio 3:7, 31%); Rf 0.61 (AcOEt/MeOH 9/1); ^1^H NMR (CDCl_3_): 4.73 (m, 2H), 3.91 (m, 2H), 3.77–3.73 (m, 2H), 3.65–3.60 (m, 4H), 3.48–3.36 (m, 4H), 1.60–1.53 (m, 4H), 1.31–1.25 (m, 42H), 0.87 (m, 6H, CH_3_) ^13^C NMR (CDCl_3_): δ 100.2, 98.9, 73.3, 73.1, 72.9, 71.9, 71.7, 71.4, 69.6, 68.0, 32.0–22.8 (CH_2_ alkyl chain), 18.0, 17.8, 14.3.

##### Tetradecyl α/β-l-Rhamnopyranoside 

White solid (β/α ratio 2:8, 33%); Rf 0.64 (AcOEt/MeOH 9/1); ^1^H NMR (CDCl_3_): 4.73 (m, 2H), 3.90 (m, 2H), 3.76–3.72 (m, 2H), 3.66–3.58 (m, 4H), 3.41–3.34 (m, 4H), 1.57–1.53 (m, 4H), 1.31–1.25 (m, 50H), 0.87 (m, 6H, CH_3_) ^13^C NMR (CDCl_3_): δ 100.1, 98.8, 73.4, 73.1, 72.1, 71.7, 71.4, 69.6, 67.9, 32.0–22.8 (CH_2_ alkyl chain), 18.1, 17.7, 14.4.

##### Hexadecyl α/β-l-rhamnopyranoside 

White solid (β/α ratio 2:8, 30%); Rf 0.68 (AcOEt/MeOH 9/1); ^1^H NMR (CDCl_3_): 4.73 (m, 2H), 3.91 (m, 2H), 3.77–3.73 (m, 2H), 3.66-3.59 (m, 4H), 3.46–3.34 (m, 4H), 1.57–1.53 (m, 4H), 1.31–1.25 (m, 58H), 0.87 (m, 6H, CH_3_) ^13^C NMR (CDCl_3_): δ 100.3, 99.7, 73.4, 72.5, 71.5, 71.0, 69.8, 68.2, 68.0, 31.0–21.9 (CH_2_ alkyl chain), 17.7, 17.5, 14.3.

#### 4.1.2. Rhamnose Esters

Acyl chloride (30.5 mmol, 1 eq.) was added dropwise to a solution of l-rhamnose (5.0 g, 30.5 mmol, 1 eq.) and DMAP (4.5 g, 36.5 mmol, 1.2 eq.) in DMF (50 mL). After 24h at 60 °C, the reaction solution was cooled. Ethyl acetate was added and the organic layer was washed with a saturated NaCl solution and HCl 1 M. The organic layer was dried with MgSO_4_ and concentrated under reduced pressure. The rhamnose ester was isolated after purification by column chromatography (EtOAc/Petroleum spirit 5:5).

##### Dodecanoyl α/β-l-Rhamnopyranoside

Yellowish oil (β/α ratio 3:7, 52%); Rf 0.48 (AcOEt/MeOH 9/1); ^1^H NMR (CDCl_3_): 5.09–4.77 (m, 2H), 4.06–3.87 (m, 4H), 3.66–3.59 (m, 2H), 3.48–3.41 (m, 2H), 2.40–2.33 (m, 4H), 1.65–1.58 (m, 4H), 1.30–1.25 (m, 38H), 0.92 (m, 6H, CH_3_) ^13^C NMR (CDCl_3_): δ 174.7, 172.0, 93.5, 92.8, 75.9, 74.2, 73.4, 72.6, 71.3, 69.9, 68.7, 68.1, 34.4, 34,3, 32.0-21.6 (CH_2_ alkyl chain), 17.6, 17.4, 13.6.

#### 4.1.3. Rhamnose Carbamates

Acyl isocyanate (12.2 mmol, 1 eq.) was added dropwise to a stirred solution of l-rhamnose (2.0 g, 12.2 mmol, 1 eq.) and NaOH (0.5 g, 12.2 mmol, 1 eq.) in water/isopropanol 1:1, wt (10 mL). After 5 h at room temperature, the reaction was neutralized with a 1 M aqueous HCl solution and the crude mixture evaporated to dryness. The rhamnose carbamate was isolated after purification by column chromatography (EtOAc/Petroleum spirit 9:1).

##### α/β-l-Rhamnopyranosyl *N*-Dodecylcarbamate

Amorphous solid (β/α ratio 5:5, 48%); Rf 0.52 (AcOEt/MeOH 9/1); ^1^H NMR (CDCl_3_): 4.97–4.91 (m, 2H), 3.59–-3.46 (m, 6H), 3.23 (m, 3H), 2.95 (m, 3H), 1.38 (m, 4H), 1.24 (m, 34H), 1.12 (m, 4H) 0.88 (m, 6H, CH_3_) ^13^C NMR (CDCl_3_): δ 154.3, 93.8, 71.5, 70,3, 70.2, 69.8, 41.8, 40.9, 31.2–22.0 (CH_2_ alkyl chain), 19.1, 17.8, 13.9.

#### 4.1.4. Mono-rhamnosyl (alkenyl) Succinates

(Alkenyl)succinic anhydride (12.2 mmol, 1 eq.) was added dropwise to a stirred solution of l-rhamnose (2.0 g, 12.2 mmol, 1 eq.) and DMAP (1.8 g, 14.6 mmol, 1.2 eq.) in DMF (20 mL). After 16 h at 60 °C, the reaction was cooled and H_2_SO_4_ 10% wt added to reach pH 5. Ethyl acetate was added and the organic phase washed with a saturated NaCl solution and HCl 1 M. The organic layer was dried with MgSO4 and concentrated under reduced pressure. The rhamnose succinate was isolated after purification by column chromatography (EtOAc).

##### Dodecenylsuccinate α/β-l-Rhamnopyranoside

Colorless oil (β/α ratio 2:8, 54%); Rf 0.53 (AcOEt/MeOH 9/1); ^1^H NMR (CDCl_3_): 5.50 (m, 2H), 5.34 (m, 2H), 5.18–5.10 (m, 2H), 4.08–4.02 (m, 2H), 3.70–3.64 (m, 4H), 3.45–3.42 (m, 4H), 2.90–2.85 (m, 2H), 2.70–2.62 (m, 4H), 2.24–1.99 (m, 8H), 1.32–1.08 (m, 34H), 0.92 (m, 6H, CH_3_) ^13^C NMR (CDCl_3_): δ 178.1, 175.5, 174.8, 134.7, 125.1, 94.5, 91.0, 74.2, 73,9, 73.4, 71.1, 70.8, 44.3, 31.7–21.9 (CH_2_ alkyl chain), 16.9, 16.4, 13.9.

### 4.2. Plant Material and Growth Conditions

Tomato seeds (*Solanum lycopersicum* L var. Ailsa craig, Scotland) were planted in pots filled with unsterilized peat moss (Gramoflor GmbH, Vechta, Germany). The plant seedlings were grown under growth chamber conditions with white fluorescent light (200 µmol/m^−2^ s^−1^), 16 h/8 h day/night photoperiod, 60% relative humidity and a temperature of 24/20 °C during four weeks before treatment.

### 4.3. Microorganism

*Botrytis cinerea* Pers. T4 (obtained from Dr. Y. Brygoo, INRA, Versailles, France) (Genbank accessions FQ790245–FQ790362) was cultivated in liquid medium composed of 250 µL of glycerol stock in 25 mL of growth medium (KH_2_PO_4_ 1.75 g/L, MgSO_4_ 0.75 g/L, Glc 4g/L, peptone 4 g/L, Tween 20 0.02% [*v*/*v*]) under controlled conditions (20 °C, continuous light, shaking at 140 rpm). A two week-old pellet of *B. cinerea* was ground and grown on solid tomato medium [25% (*v*/*v*) tomato juice and 2.5% (*w*/*v*) agar, 5.5 < pH < 6] during two weeks at 22 °C. Collected conidia were resuspended in Potato Dextrose Broth (PDB) to a final density of 10^5^ conidia mL^−1^. After incubation for 3 h at 22 °C and 140 rpm, germinated spores were used for plant inoculation by depositing a droplet onto the adaxial surface of the primary leaflets.

### 4.4. ROS Production

Reactive oxygen species (ROS) production was analyzed on tomato leaf disks as described by Smith et al. (2014) with some modifications. Briefly, peroxidase (Peroxidase from Horseradish, HRP Sigma, Darmstadt, Germany) was prepared as a 500× stock solution by dissolving 10 mg mL^−1^ in sterile H_2_O. Aliquots of 10–30 μL were stored at −20 °C and used at a final concentration of 20 μg ml^-1^. A 500× luminol stock solution was also prepared by dissolving 17 mg luminol (luminol sodium salt, Sigma Darmstadt, Germany) in 1 mL of ultrapure water and used at a final concentration of 0.2 μM. The stock solution was wrapped in aluminum foil and renewed every day. One day before ROS assay, leaf disks of 6 mm diameter were cut from 4-week-old tomato plants. Each leaf disk was placed in an individual well of a 96-well microplate (Optiplate TM-96, white, 96-well, PerkinElmer, Walthman, MA, USA) containing 150 μL of ultrapure water and then incubated at room temperature for 24 h to reduce the wounding response (Luzuriaga-Loaiza et al., 2018) [21]. The elicitation solution was prepared containing luminol and HRP and tested smRLs at 100 µM. Flagellin (Flg22 peptide at 1 µM) and dimethyl sulfoxide (DMSO, 0.1%) were used as positive and negative controls, respectively. All solutions were kept at room temperature. Immediately prior to elicitation, the incubating water solution was carefully removed from each well, avoiding any tissue damage or desiccation. Then, 150 μL of the elicitation solution was quickly added to each well containing a leaf disc. For luminol-based ROS production, the plate was placed without delay into TECAN (Infinite F200 Pro Luminometer, Männedorf, Switzerland) to measure ROS production between 0 and 12 h. The means ± standard deviations originated from three independent experiments realized in duplicates, each replicate consisted of a pool of six plantlets.

### 4.5. Antifungal Activity

#### 4.5.1. Conidial Germination Assay

*B.cinerea* conidia germination assay was done as described by Sanchez et al. (2012) [33] with some modifications. This assay was conducted in sterile flat-bottom 96-well microplates. In each well, a volume of 100 µL containing 5 × 10^4^ conidia ml^−1^ in PDB liquid medium (24 g L^−1^) and each smRLs solution at 100, 200 and 300 µM was poured. DMSO (0.1%) as a negative control was similarly used. Germ tube growth was observed using inverted light microscopy (Leica, Wetzlar, Germany) at 8 and 24 h.

#### 4.5.2. Fungal Development Assay

Conidia of *B. cinerea* were collected by adding 10 mL of sterile water on the surface of the medium and gently scratching the mycelium with a sterile scalpel. Conidia suspension was determined with a Fushs-Rosenthal hemacytometer (Paul Marienfeld GmbH & Co. KG, Lauda-Königshofen, Germany) and adjusted to 2.5 × 10^3^ conidia mL^−1^. Antifungal assays were conducted in 96-microplate wells (96-wells-F, sterile, VWR, Radnor, PA, USA). Each well contained 45 μL of 0.1% filter-sterilized DMSO (as control) or 45 µL of each filter-sterilized smRLs at 100, 200 or 300 µM in 105 µL of sterile solid PDA medium. Each plate contained a minimum of 8 replicates (wells) of 0.1% DMSO as control and 8 replicates of each smRL concentration. smRLs were left to diffuse for one night at 4 °C in the dark, prior to adding 40 μL of the conidia suspension in each well. The microplates were incubated at 25 °C in the dark. Absorbance was measured regularly until the controls on 0.1% DMSO reached 1.0 ± 0.1 at 620 nm or ceased to grow, using a microplate absorbance reader (Multiskan bichromatic, Thermo Labsystems, Helsinki, Finland). Each well was considered as an experimental unit. Percentage of growth inhibition following smRLs addition prior to the pathogen was calculated as follows: %inhibition = [(Abs control dx − Abs control d0) − (Abs rhamnolipid dx − Abs rhamnolipid d0)]/(Abs control dx − Abs control d0) *100, where: Abs: absorbance, d0: absorbance at time 0 (day 0), dx: absorbance at day x, control: 0.1%, DMSO control. Inhibition of conidial germination or mycelium development was checked under a compound microscope (Olympus BH2, Olympus Optical, GmbH, Hamburg, Germany) at 20–40× magnifications.

#### 4.5.3. Dual-Test Assay

To examine the antagonistic effects of smRLs against the actively growing *B. cinerea*, discs of solid medium tomato juice [25% (*v*/*v*) tomato juice and 2.5% (*w*/*v*) agar, 5.5 < pH < 6] (5 mm diam.) were taken from the edge of actively growing *B. cinerea* mycelium and placed on the surface of a fresh PDA medium in Petri plates. The plug was placed upside down in the middle of the Petri plates and further incubated at 25 °C in the dark. After 1 day of growth, 3 mL of each filter-sterilized smRL solution was added onto the surface of the Petri plates and further incubated under the same conditions as above. Growth evaluation was assessed every 24 h during 3 days until pathogen growth in the absence of smRL (0.1% DMSO control) completely covered the late or ceased growth. Five Petri plates constituted an experimental unit for each treatment and smRL concentration. Every plate was photographed and the area covered by the fungus was estimated using the ImageJ software (National Institutes of Health, Bethesda, MD, USA).

### 4.6. Protection Test

Four-week-old tomato plants (3 plants/condition, 4 leaves/plant) were sprayed with each smRL solution (1 mL) at 300 µM. Plants were also treated with DMSO (0.1%) as a negative control. Three plants were considered for each condition. The plants were placed in a culture room under white fluorescent light (200 µmol/m^−2^ s^−1^), 16 h/8 h day/night photoperiod and a temperature of 24/20 °C. After 3 days, four leaves per plant were collected and deposited in Petri plates containing water-soaked Whatman paper. The abaxial surface of the leaves were deposited in contact with Wathman paper. Then, 10 μL of a conidial suspension (1 × 10^5^ conidia mL^−1^) of *B. cinerea* was dropped onto the adaxial surface of the leaves. As a control, leaves were mock-inoculated with PDB. After (mock) inoculation, the leaves were placed in a growth chamber under controlled conditions (20 °C, continuous light, and humidity of 100%). The diameter of each lesion was measured 72 and 96 h after inoculation (hai). Three independent experiments at three different times were considered.

### 4.7. RNA Extraction, cDNA Synthesis, and Real-Time PCR

For gene expression, plants were grown and treated as described in the paragraph “protection test” and were infected or not with *B. cinerea*. The leaves were collected 48 h after infection and conserved in liquid nitrogen. Total RNAs were extracted from 50 mg of each sample, using Extract-All reagent (Eurobio, Les Ulis, France). Reverse transcription was performed from 500 µg of RNA using the Verso cDNA synthesis kit (Thermo Fisher Scientific, Walthman, MA, USA) according to the manufacturer′s instructions. The transcription levels were determined as described by Issa et al. (2018) by qPCR using the CFX 96TM real-time system (Bio-Rad, Hercules, CA, USA) and the combined SYBR Green Master PCR kit as recommended by the manufacturer (Applied Biosystems, Foster City, CA, USA). PCR reactions were conducted in duplicate in 96-well plates (15 µL per well) in a buffer containing 1× SYBR Green I mix (including Taq polymerase, dNTPs, and SYBR Green dye), forward and reverse primers 280 nM, and 1:10 dilution of reverse transcription RNA. After denaturation at 95 °C for 15 min, the amplification occurred in a two-step procedure: 15 s of denaturation at 95 °C and 1 min of annealing/extension at 60 °C, for a total of 30 cycles. Specific primers used are listed in Supplementary Materials (Table S2). EF1α and ACT were used as internal controls. The reference sample consisted of “0.1% DMSO control” sample, chosen to represent 1× expression of the gene of interest.

### 4.8. Statistical Analysis

Effect of smRLs on in vitro germination of spores and subsequent growth of *B. cinerea* in wells of microplates were analysed by an analysis of variance followed by Tukey′s multiple comparison test (*p* ≤ 0.05), and their effect on in vitro radial growth of *B. cinerea* in Petri plates were analysed by a repeated-measures analysis of variance followed by Tukey’s multiple comparison test (*p* ≤ 0.05), using the JMP 14.0 software (SAS Institute Inc., Cary, NC, USA). For experiments targeting smRLs effect on protection and relative defense gene expression, statistical analyses were carried out by analysis of variance followed by Turkey’s multiple comparison test (*p* ≤ 0.05), using R Commander. For microplates data, statistical analyses were led on the difference of optical densities between ‘time x’ minus ‘time 0′. Data that did not assume the normality were root-transformed before analysis.

## Supplementary Materials

The Supplementary Materials are available online. Figure S1: Production of ROS after treatment with Eth-smRLs exhibiting different carbon chain. lengths; Flagellin (Flg22 peptide at 1 μM) and DMSO (0.1%) were used as positive and negative controls, respectively. Reactive oxygen species (ROS) production was measured using the chemiluminescence of luminol and photon counts were expressed as relative luminescence units 8 (RLUs). Figure S2: smRLs dose response (25, 50, and 50 µM) on ROS production in tomato leaf disks. DMSO (0.1%) was used as control. Reactive oxygen species (ROS) production was measured using the chemiluminescence of luminol and photon counts were expressed as relative luminescence units (RLUs). Table S1: List of Synthetic mono-rhamnolipids used in this study. Table S2: List of primer pairs used in qRT-PCR experiments.

## References

[1] Cook D.E., Mesarich C.H., Thomma B.P. (2015). Understanding plant immunity as a surveillance system to detect invasion. Annu. Rev. Phytopathol..

[2] Kanyuka K., Rudd J.J. (2019). Cell surface immune receptors: The guardians of the plant’s extracellular spaces. Curr. Opin. Plant. Biol..

[3] Schellenberger R., Touchard M., Clement C., Baillieul F., Cordelier S., Crouzet J., Dorey S. (2019). Apoplastic invasion patterns triggering plant immunity: Plasma membrane sensing at the frontline. Mol. Plant. Pathol..

[4] van der Burgh A.M., Joosten M. (2019). Plant immunity: Thinking outside and inside the box. Trends Plant. Sci..

[5] Bigeard J., Colcombet J., Hirt H. (2015). Signaling mechanisms in pattern-triggered immunity (PTI). Mol. Plant..

[6] Yip Delormel T., Boudsocq M. (2019). Properties and functions of calcium-dependent protein kinases and their relatives in *Arabidopsis thaliana*. New Phytol..

[7] Garcia-Brugger A., Lamotte O., Vandelle E., Bourque S., Lecourieux D., Poinssot B., Wendehenne D., Pugin A. (2006). Early signaling events induced by elicitors of plant defenses. Mol. Plant. Microbe Interact..

[8] Pieterse C.M., Van der Does D., Zamioudis C., Leon-Reyes A., Van Wees S.C. (2012). Hormonal modulation of plant immunity. Annu. Rev. Cell. Dev. Biol..

[9] Boller T., Felix G. (2009). A renaissance of elicitors: Perception of microbe-associated molecular patterns and danger signals by pattern-recognition receptors. Annu. Rev. Plant. Biol..

[10] Boutrot F., Zipfel C. (2017). Function, discovery, and exploitation of plant pattern recognition receptors for broad-spectrum disease resistance. Annu. Rev. Phytopathol..

[11] Bektas Y., Eulgem T. (2014). Synthetic plant defense elicitors. Front. Plant. Sci..

[12] Gorlach J., Volrath S., Knauf-Beiter G., Hengy G., Beckhove U., Kogel K.H., Oostendorp M., Staub T., Ward E., Kessmann H. (1996). Benzothiadiazole, a novel class of inducers of systemic acquired resistance, activates gene expression and disease resistance in wheat. Plant. Cell.

[13] Lawton K.A., Friedrich L., Hunt M., Weymann K., Delaney T., Kessmann H., Staub T., Ryals J. (1996). Benzothiadiazole induces disease resistance in *Arabidopsis* by activation of the systemic acquired resistance signal transduction pathway. Plant. J..

[14] Uknes S., Mauch-Mani B., Moyer M., Potter S., Williams S., Dincher S., Chandler D., Slusarenko A., Ward E., Ryals J. (1992). Acquired resistance in *Arabidopsis*. Plant. Cell.

[15] Ward E.R., Uknes S.J., Williams S.C., Dincher S.S., Wiederhold D.L., Alexander D.C., Ahl-Goy P., Metraux J.P., Ryals J.A. (1991). Coordinate gene activity in response to agents that induce systemic acquired resistance. Plant. Cell.

[16] Rodriguez-Salus M., Bektas Y., Schroeder M., Knoth C., Vu T., Roberts P., Kaloshian I., Eulgem T. (2016). The synthetic eicitor 2-(5-bromo-2-hydroxy-phenyl)-thiazolidine-4-carboxylic acid links plant immunity to hormesis. Plant. Physiol..

[17] Knoth C., Salus M.S., Girke T., Eulgem T. (2009). The synthetic elicitor 3,5-dichloroanthranilic acid induces NPR1-dependent and NPR1-independent mechanisms of disease resistance in *Arabidopsis*. Plant. Physiol..

[18] Bektas Y., Rodriguez-Salus M., Schroeder M., Gomez A., Kaloshian I., Eulgem T. (2016). The synthetic elicitor DPMP (2,4-dichloro-6-{(E)-[(3-methoxyphenyl)imino]methyl}phenol) triggers strong immunity in *Arabidopsis thaliana* and tomato. Sci. Rep..

[19] Brotman Y., Makovitzki A., Shai Y., Chet I., Viterbo A. (2009). Synthetic ultrashort cationic lipopeptides induce systemic plant defense responses against bacterial and fungal pathogens. Appl. Environ. Microbiol..

[20] Cambiagno D.A., Lonez C., Ruysschaert J.M., Alvarez M.E. (2015). The synthetic cationic lipid diC14 activates a sector of the *Arabidopsis* defence network requiring endogenous signalling components. Mol. Plant. Pathol..

[21] Luzuriaga-Loaiza P., Schellenberger R., De Gaetano Y., Obounou Akong F., Villaume S., Crouzet J., Haudrechy A., Baillieul F., Clément C., Lins L. (2018). Synthetic rhamnolipid bolaforms trigger an innate immune response in *Arabidopsis thaliana*. Sci. Rep..

[22] Hogan D.E., Tian F., Malm S.W., Olivares C., Palos Pacheco R., Simonich M.T., Hunjan A.S., Tanguay R.L., Klimecki W.T., Polt R. (2019). Biodegradability and toxicity of monorhamnolipid biosurfactant diastereomers. J. Hazard. Mater..

[23] Johann S., Seiler T.-B., Tiso T., Bluhm K., Blank L.M., Hollert H. (2016). Mechanism-specific and whole-organism ecotoxicity of mono-rhamnolipids. Sci. Total Environ..

[24] Mohan P.K., Nakhla G., Yanful E.K. (2006). Biokinetics of biodegradation of surfactants under aerobic, anoxic and anaerobic conditions. Water Res..

[25] Borah S.N., Goswami D., Sarma H.K., Cameotra S.S., Deka S. (2016). Rhamnolipid biosurfactant against *Fusarium verticillioides* to control stalk and ear rot disease of maize. Front. Microbiol..

[26] Goswami D., Handique P.J., Deka S. (2014). Rhamnolipid biosurfactant against *Fusarium sacchari* the causal organism of pokkah boeng disease of sugarcane. J. Basic Microbiol..

[27] Kim B.S., Lee J.Y., Hwang B.K. (2000). In vivo control and in vitro antifungal activity of rhamnolipid B, a glycolipid antibiotic, against *Phytophthora capsici* and *Colletotrichum orbiculare*. Pest Manage. Sci..

[28] Monnier N., Furlan A.L., Buchoux S., Deleu M., Dauchez M., Rippa S., Sarazin C. (2019). Exploring the dual interaction of natural rhamnolipids with plant and fungal biomimetic plasma membranes through biophysical studies. Int. J. Mol. Sci..

[29] Stanghellini M.E., Miller R.M. (1997). Biosurfactants: Their identity and potential efficacy in the biological control of zoosporic plant pathogen. Plant. Dis..

[30] Yan F., Hu H., Lu L., Zheng X. (2016). Rhamnolipids induce oxidative stress responses in cherry tomato fruit to *Alternaria alternata*. Pest. Manage. Sci..

[31] Monnier N., Cordier M., Dahi A., Santoni V., Guenin S., Clément C., Sarazin C., Penaud A., Dorey S., Cordelier S. (2020). Semipurified rhamnolipid mixes protect *Brassica napus* against *Leptosphaeria maculans* early infections. Phytopathology.

[32] Monnier N., Furlan A., Botcazon C., Dahi A., Mongelard G., Cordelier S., Clément C., Dorey S., Sarazin C., Rippa S. (2018). Rhamnolipids from *Pseudomonas aeruginosa* are elicitors triggering *Brassica napus* protection against *Botrytis cinerea* without physiological disorders. Front. Plant. Sci..

[33] Sanchez L., Courteaux B., Hubert J., Kauffmann S., Renault J.H., Clément C., Baillieul F., Dorey S. (2012). Rhamnolipids elicit defense responses and induce disease resistance against biotrophic, hemibiotrophic, and necrotrophic pathogens that require different signaling pathways in *Arabidopsis* and highlight a central role for salicylic acid. Plant. Physiol..

[34] Varnier A.L., Sanchez L., Vatsa P., Boudesocque L., Garcia-Brugger A., Rabenoelina F., Sorokin A., Renault J.H., Kauffmann S., Pugin A. (2009). Bacterial rhamnolipids are novel MAMPs conferring resistance to *Botrytis cinerea* in grapevine. Plant. Cell Environ..

[35] Das R., Mukhopadhyay B. (2016). Chemical O-Glycosylations: An overview. ChemistryOpen.

[36] Hricovíniová Z., Hricovíni M. (2014). An efficient synthesis of novel l-rhamnose based non-ionic surfactants under controlled microwave irradiation. Tetrahedron: Asymmetry.

[37] Zheng H., Singh N., Shetye G.S., Jin Y., Li D., Luk Y.Y. (2017). Synthetic analogs of rhamnolipids modulate structured biofilms formed by rhamnolipid-nonproducing mutant of *Pseudomonas aeruginosa*. Bioorg. Med. Chem..

[38] Houlmont J.-P., Rico-Lattes I., Perez E., Bordat P. (2005). Medicament Comprising a Reducing Alkyl-Sugar Monomer for the Treatment of Inflammatory Disorders. France Patent.

[39] Mizutani T. (1985). Protective effects of butylated hydroxyanisole and its analogs on the lung toxicity of butylated hydroxytoluene in mice. Res. Commun. Chem. Pathol. Pharmacol..

[40] Raposo C.D., Petrova K.T., Barros M.T., Calhelha R.C., Sokovic M., Ferreira I.C. (2016). Synthesis, characterization, antimicrobial and antitumor activities of sucrose Octa(N-ethyl)carbamate. Med. Chem..

[41] Christian D., Fitremann J., Bouchu A., Queneau Y. (2004). Preparation of amphiphilic sucrose carbamates by reaction with alkyl isocyanates in water–alcohol mixtures. Tetrahedron Lett..

[42] Le Guenic S., Chaveriat L., Lequart V., Joly N., Martin P. (2019). Renewable surfactants for biochemical applications and nanotechnology. J. Surfactants Deterg..

[43] Vatsa P., Sanchez L., Clément C., Baillieul F., Dorey S. (2010). Rhamnolipid biosurfactants as new players in animal and plant defense against microbes. Int. J. Mol. Sci..

[44] Wang H., Williams P.A., Senan C. (2014). Synthesis, characterization and emulsification properties of dodecenyl succinic anhydride derivatives of gum Arabic. Food Hydrocoll..

[45] Zhou J., Ren L., Tong J., Xie L., Liu Z. (2009). Surface esterification of corn starch films: Reaction with dodecenyl succinic anhydride. Carbohydr. Polym..

[46] Issa A., Esmaeel Q., Sanchez L., Courteaux B., Guise J.F., Gibon Y., Ballias P., Clement C., Jacquard C., Vaillant-Gaveau N. (2018). Impacts of *Paraburkholderia phytofirmans* strain PsJN on tomato (*Lycopersicon esculentum* L.) under high temperature. Front. Plant. Sci..

[47] Menhour B., Mayon P., Plé K., Bouquillon S., Dorey S., Clément C., Deleu M., Haudrechy A. (2015). A stereocontrolled synthesis of the hydrophobic moiety of rhamnolipids. Tetrahedron Lett..

[48] Felix G., Duran J.D., Volko S., Boller T. (1999). Plants have a sensitive perception system for the most conserved domain of bacterial flagellin. Plant. J..

[49] Nasir M.N., Crowet J.M., Lins L., Obounou Akong F., Haudrechy A., Bouquillon S., Deleu M. (2016). Interactions of sugar-based bolaamphiphiles with biomimetic systems of plasma membranes. Biochimie.

[50] Henry G., Deleu M., Jourdan E., Thonart P., Ongena M. (2011). The bacterial lipopeptide surfactin targets the lipid fraction of the plant plasma membrane to trigger immune-related defence responses. Cell. Microbiol..

[51] Jourdan E., Henry G., Duby F., Dommes J., Barthelemy J.P., Thonart P., Ongena M. (2009). Insights into the defense-related events occurring in plant cells following perception of surfactin-type lipopeptide from *Bacillus subtilis*. Mol. Plant.-Microbe Interact..

[52] Steinberg G., Schuster M., Gurr S.J., Schrader T.A., Schrader M., Wood M., Early A., Kilaru S. (2020). A lipophilic cation protects crops against fungal pathogens by multiple modes of action. Nature Commun..

[53] Goswami D., Borah S.N., Lahkar J., Handique P.J., Deka S. (2015). Antifungal properties of rhamnolipid produced by *Pseudomonas aeruginosa* DS9 against *Colletotrichum falcatum*. J. Basic Microbiol..

[54] Rodrigues A.I., Gudina E.J., Teixeira J.A., Rodrigues L.R. (2017). Sodium chloride effect on the aggregation behaviour of rhamnolipids and their antifungal activity. Sci. Rep..

[55] Sha R., Meng Q. (2016). Antifungal activity of rhamnolipids against dimorphic fungi. J. Gen. Appl. Microbiol..

