# Supplementary material for: Synthetic Mono-Rhamnolipids Display Direct Antifungal Effects and Trigger an Innate Immune Response in Tomato against Botrytis Cinerea
Source: Molecules. 2020 Jul 8;25(14):3108. doi: 10.3390/molecules25143108 (PMC7397090; doi:10.3390/molecules25143108)
Supplement: Supplementary file 1 [file molecules-25-03108-s001.pdf]

molecules

MDPI

Supplemental data

Molecular formula	Synonyms	Structural formula
Eth-C4	Butyl *α*/β-L-rhamnopyranoside	
Eth-C6	Hexyl *α*/β-*L*-rhamnopyranoside	
Eth-C8	Octyl *α*/β-*L*-rhamnopyranoside	
Eth-C10	Decyl *α*/β-L-rhamnopyranoside	
Eth-C12	Dodecyl *α*/β-L-rhamnopyranoside	
Eth-C14	Tetradecyl *α*/β-L-rhamnopyranoside	
Eth-C16	Hexadecyl *α*/β-L-rhamnopyranoside	
Eth-C18	Octadecyl *α*/β-L-rhamnopyranoside	
Est-C12	Dodecanoyl *α*/β-*L*-rhamnopyranoside	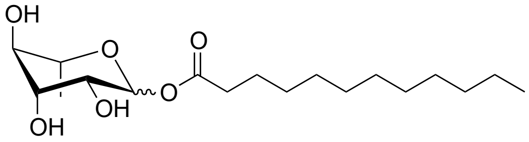
Car-C12	*α*/β-L-Rhamnopyranosyl *N*-dodecylcarbamate	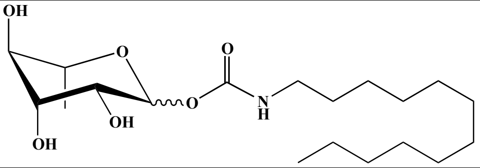
Suc-C12	Dodecenylsuccinate *α*/β-L-rhamnopyranoside	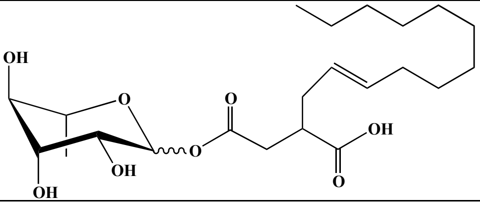

Table S1: List of Synthetic mono-rhamnolipids used in this study

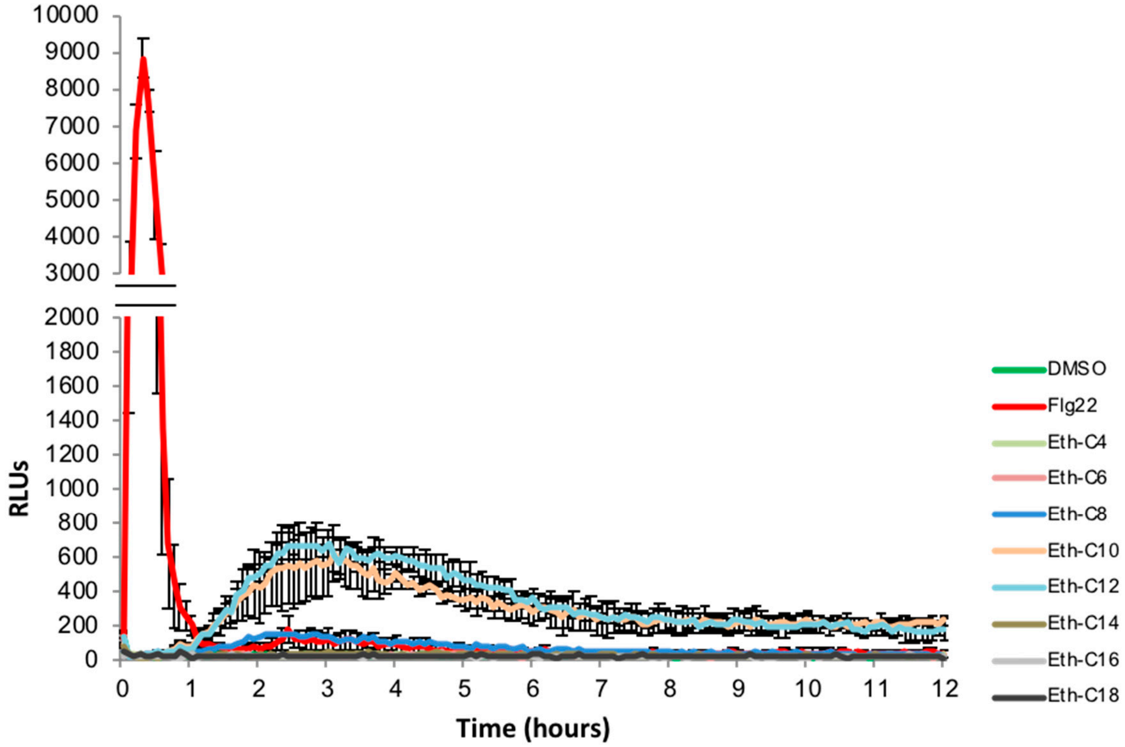

Figure S1. Production of ROS after treatment with Eth-smRLs exhibiting different carbon chain lengths; Flagellin (Flg22 peptide at 1 μM) and DMSO (0.1%) were used as positive and negative controls, respectively. Reactive oxygen species (ROS) production was measured using the chemiluminescence of luminol and photon counts were expressed as relative luminescence units (RLUs).

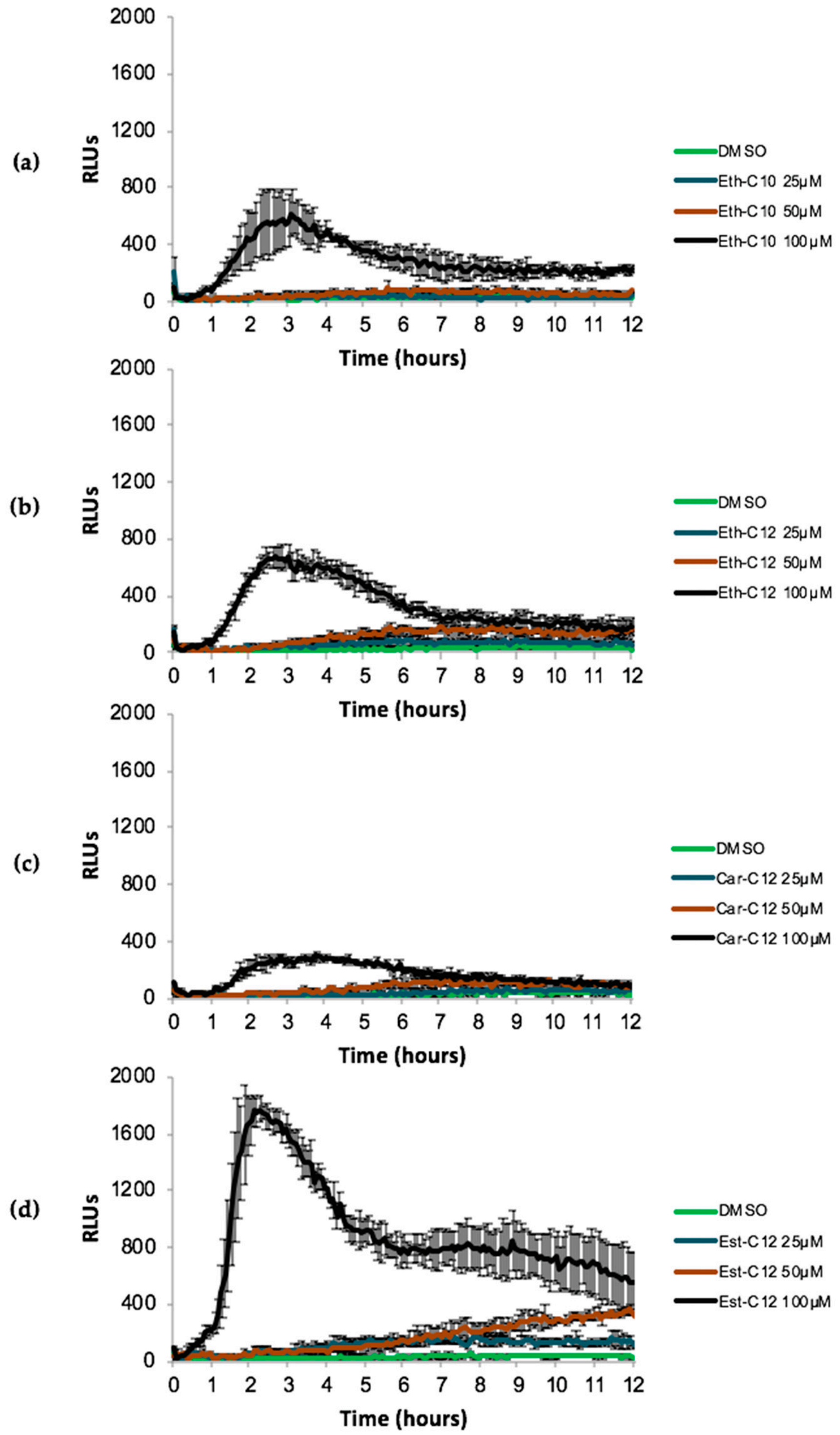

1.

Figure S2. smRLs dose–response (25, 50, and 50 μM) on ROS production in tomato leaf disks. DMSO (0.1%) was used as control. Reactive oxygen species (ROS) production was measured using the chemiluminescence of luminol and photon counts were expressed as relative luminescence units (RLUs).
